# Chemical Constituents and Biological Activities of *Croton heliotropiifolius* Kunth

**DOI:** 10.3390/antibiotics10091074

**Published:** 2021-09-05

**Authors:** Priscilla Augusta de Sousa Fernandes, Josefa Carolaine Pereira da Silva, Débora Lima Sales, Paulo Riceli Vasconcelos Ribeiro, Edy Sousa de Brito, Marta Regina Kerntopf, Gyllyandeson de Araújo Delmondes, Jacqueline Cosmo Andrade Pinheiro, Gerson Javier Torres Salazar, Francisco Lucas Alves Batista, Francisco Ernani Alves Magalhães, Maria Celeste Vega Gomez, Míriam Rolón, Cathia Coronel, Jaime Ribeiro-Filho, José Weverton Almeida-Bezerra, Abolghasem Siyadatpanah, Veeranoot Nissapatorn, Maria de Lourdes Pereira, Henrique Douglas Melo Coutinho, Maria Flaviana Bezerra Morais-Braga

**Affiliations:** 1 Department of Biological Chemistry, Universidade Regional do Cariri—URCA, Av. Cel Antônio Luis, 1161, Pimenta, Crato 63105-010, Brazil; priscilla.fernandes@urca.br (P.A.d.S.F.); debora.lima.sales@gmail.com (D.L.S.); martareginakerntopfm@outlook.com (M.R.K.); gyllyandesondelmondes@gmail.com (G.d.A.D.); timotygertor@yahoo.com (G.J.T.S.); lucas-a-b-181@hotmail.com (F.L.A.B.); 2 Department of Biological Sciences, Universidade Regional do Cariri—URCA, Av. Cel Antônio Luis, 1161, Pimenta, Crato 63105-010, Brazil; carol.bio1881@outlook.com (J.C.P.d.S.); flavianamoraib@yahoo.com.br (M.F.B.M.-B.); 3 Embrapa Agroindústria Tropical, Tropical R. Pernambuco, 2270–Pici, Fortaleza 60511-110, Brazil; paulo.riceli@embrapa.br (P.R.V.R.); edy.brito@embrapa.br (E.S.d.B.); 4 Laboratório de Bioensaios, Universidade Federal do Cariri—UFCA, R. Olegário Emídio de Araújo, s/n, Centro, Brejo Santo 63260-000, Brazil; andradejacquelinec@gmail.com; 5 Inhamuns Region Education, Science and Technology Center, Universidade Estadual do Ceará—UECE, BR-020—Bezerra de Sousa, Tauá 63660-000, Brazil; fernanimagalhaes@yahoo.com.br; 6 Center for the Development of Scientific Research, Asuncion 1255, Paraguay; mcvegagomez@gmail.com (M.C.V.G.); rolonmiriam@gmail.com (M.R.); 7 Laboratório de Investigação em Genética e Hematologia Translacional, Instituto Gonçalo Moniz, FIOCRUZ, Salvador 40296-710, Brazil; cathiacoronel@gmail.com (C.C.); jaimeribeirofilho@gmail.com (J.R.-F.); 8 Department of Botany, Universidade Federal de Pernambuco—UFPE, Av. Prof. Moraes Rego, 1235, Recife 50670-901, Brazil; weverton.almeida@urca.br; 9 Ferdows School of Paramedical and Health, Birjand University of Medical Sciences, Birjand 9717853577, Iran; asiyadatpanah@yahoo.com; 10 School of Allied Health Sciences, World Union for Herbal Drug Discovery (WUHeDD) and Research Excellence Center for Innovation and Health Products (RECIHP), Walailak University, Nakhon Si Thammarat 80160, Thailand; veeranoot.ni@wu.ac.th; 11 CICECO-Aveiro Institute of Materials and Department of Medical Sciences, University of Aveiro, 3810-193 Aveiro, Portugal

**Keywords:** *Croton heliotropiifolius*, phenolic compounds, antioxidant, antifungal, cytotoxicity

## Abstract

*Croton heliotropiifolius* Kunth (Euphorbiaceae), whose occurrence has already been registered in the most varied Brazilian biomes, is commonly found in the Chapada do Araripe, Ceará. The species is traditionally used to treat fungal, parasitic, and degenerative diseases. This study investigated the chemical composition and pharmacological potential (antioxidant, antifungal, antiparasitic, and cytotoxic) of an aqueous extract obtained from the roots of *C. heliotropiifolius*. Following a qualitative phytochemical screening, the chemical constituents were identified by ultra-efficiency liquid chromatography coupled witha quadrupole/time-of-flight system (UPLC-QTOF). The antioxidant potential was verified by thin-layer chromatography (TLC). The direct and combined antifungal activity of the extract against opportunistic *Candida* strains was investigated using the microdilution method. The minimal fungicidal concentration (MFC) was determined by subculture, while the modulation of the morphological transition (fungal virulence) was evaluated by light microscopy. The in vitro antiparasitic activity was analyzed using epimastigotes of *Trypanosoma cruzi* and promastigotes of *Leishmania braziliensis* and *Leishmania infantum,* while cytotoxicity was determined in cultures of mouse fibroblasts. The phytochemical analysis identified the presence of acids, terpenes, flavonoids, lignans, and alkaloids. Among these constituents, the presence of polar and non-polar phenolic compounds with known antioxidant action was highlighted. While the extract showed clinically ineffective antifungal effects, it could enhance the effectiveness of fluconazole, in addition to inhibiting the morphological transition associated with increased virulence in *Candida* strains. Although the extract showed low cytotoxicity against fibroblasts, it also had weak antiparasitic effects. In conclusion, *Croton heliotropiifolius* is a source of natural products with antifungal and antioxidant potential.

## 1. Introduction

Natural products have significantly impacted the discovery of new drugs [1], as they represent an alternative for rapid and economic development, due to their high availability [2]. In this context, ethnopharmacological research stands out for documenting the use of plants in folk medicine, serving as a pre-screen to guide pharmacological studies.

According to Patwardhan and Mashelkar [2], this drug development strategy based on natural products has been returned to a prominent place in the pharmacological market [3], despite the existence of some skepticism about the effectiveness of natural products used in folk medicine and the development of synthetic substances in recent decades [4].

In recent years, natural products have gained prominence for their therapeutic potential in the treatment of parasitic infections, such as those caused by bacteria, fungi, and protozoa. Since the rapid emergence of resistant strains has increasingly limited the effectiveness of conventional drugs, the identification of secondary metabolites of plants may represent an important alternative in the management of infections by resistant microorganisms [5,6,7,8]. 

Fungal infections caused by yeasts of the genus *Candida* stand out due to their high incidence in hospital environments [9]. In this context, *Candida albicans* presents remarkable virulence, inwhich the morphological change of the yeast to forms that present pseudo-hyphae and hyphae stand out for increasing both virulence and resistance to antifungal drugs [10,11,12,13]. 

Brazil is one of the 149 countries affected by neglected diseases, which affect more than one billion people worldwide, especially populations withsocial vulnerability and precarious access to basic sanitation. Neglected tropical diseases (NTDs), such as leishmaniasis and American trypanosomiasis (Chagas’ disease), are a group of communicable diseases with prevalence in tropical and subtropical regions, where the population is in direct contact with vectors [14]. When compared withthe context of other diseases of public health importance, investments into research, drug development, and control of NTDs are quite reduced. Since NTDs incapacitate and kill millions of people around the world [15], the development of safe and effective medicines is urgent. In this context, evidence indicates that compounds capable of modulating the inflammatory response and oxidative imbalance have potential applications in the development of new drugs for the treatment of infectious and non-communicable chronic inflammatory diseases [16,17,18].

*Croton heliotropiifolius* Kunth (Euphorbiaceae) is a shrub popularly known as “velame” and “pau-de-leite” [19]. This species is widely distributed in the Caatinga, a seasonally dry tropical forest biome located in Northeastern Brazil [20]. Ethnopharmacological studies have registered the use of this species for the treatment of diseases such as diabetes mellitus, Alzheimer’s, Parkinson’s [21], flu, pain, inflammation, skin diseases [22], back pain [23], cough, stomach pain, menstrual disorders, anemia, blood disorders [19], and parasitic diseases [24].

A recent ethnobotanical study [25] conducted in Chapada do Araripe (Ceará, Brazil) identified *C. heliotropiifolius* as a species with multiple therapeutic indications, including the treatment of symptoms resulting from urinary, intestinal, and skin infections. Together, this evidence indicates that *C. heliotropiifolius* may have antimicrobial [26], antiparasitic [27], and antioxidant [21] activities. Thus, considering the therapeutic potential suggested by ethnobotanical studies, this work aimed to determine the chemical profile and experimentally investigate the antioxidant, antifungal, antiparasitic, and cytotoxic potential of an aqueous extract obtained from the roots of *Croton heliotropiifolius*.

## 2. Results

### 2.1. Phytochemical Compositionof Croton heliotropiifolius

A preliminary phytochemical analysis of the aqueous extract of *C. heliotropiifolius* roots revealed the presence of several classes of special metabolites (Table 1), including alkaloids, phenols, xanthones, and some subclasses of flavonoids (flavones, flavonols, and flavanones). On the other hand, the phytochemical prospection did not identify the presence of coumarins, steroids, tannins, triterpenoids, or flavonoids such as anthocyanins, anthocyanidins, catechins, flavanonols, and leucoanthocyanidins.

**Figure 1:**
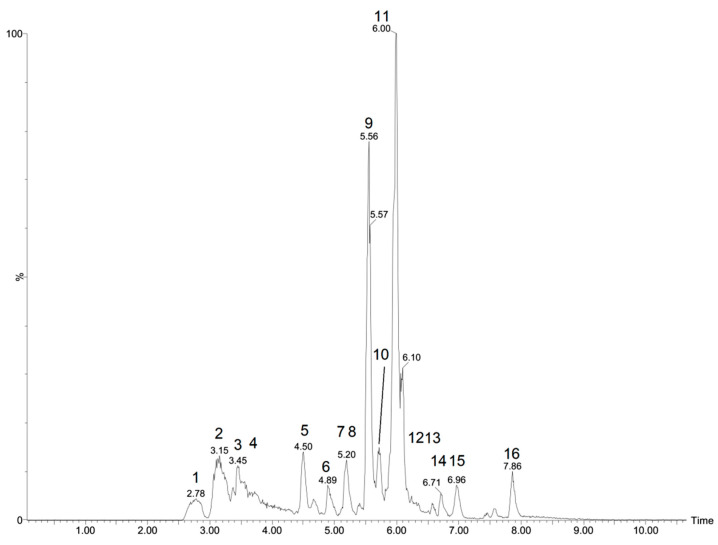
*Croton heliotropiifolius* root extract chromatogram in the negative mode.

Figure 1 shows the extract’s chromatograms in the negative mode. The characterization of the compounds is shown in Table 2, which contain full information regarding their mass, retention time, fragmentation, and error, generated by MassLynx software.

**Figure 2:**
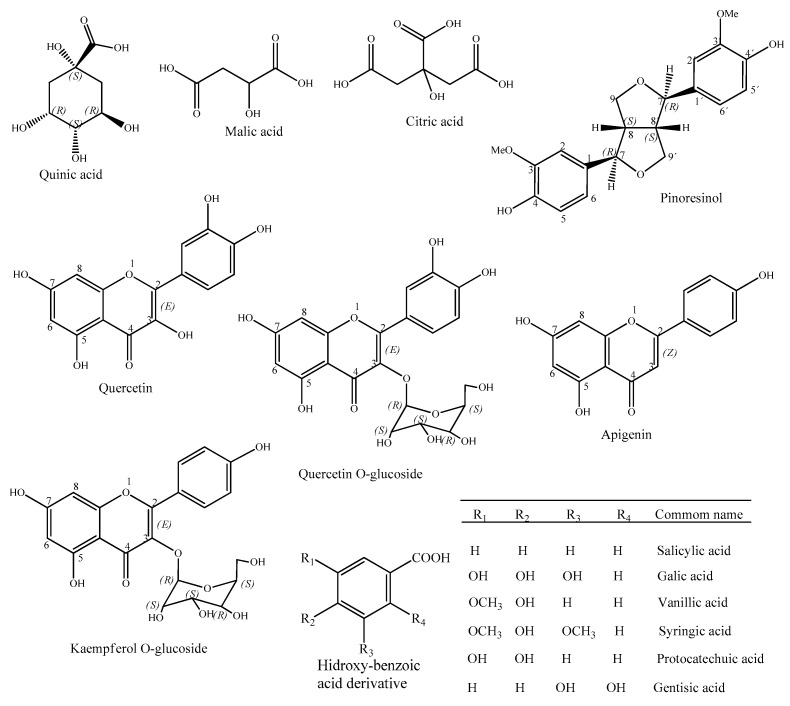
Chemical structures of the compounds identified in the negative mode.

**Figure 3:**
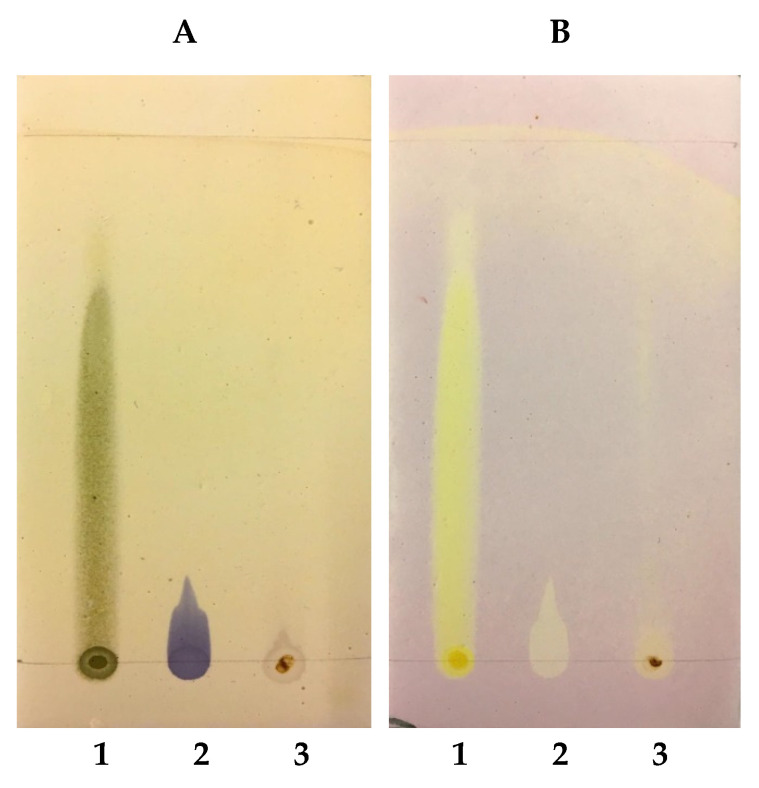
Gypsum-starch chromatoplates (1:1) from the AECHR stained with: (**A**) FeCl_3_ (detection of phenols) and (**B**) DPPH (detection of antioxidants). Quercetin (1); gallic acid (2); AECHR (3). Chloroform/ethanol (9:1) elution.

Through the analysis of the 16 peaks shown in the chromatogram in negative mode (Table 2, Figure 2), it was possible to identify nine compounds based on the literature available for the Euphorbiaceae family, including four acids (quinic, *m*/*z* 191.0549; malic, *m*/*z* 133.0141; citric, *m*/*z* 191.0185; and hydroxybenzoic acid derivative, *m*/*z* 253.0347), four flavonoids (quercetin-O-glycoside, kaempferol-O-glucoside, quercetin and apigenin), *m*/*z* 463.0894), a lignan (pinoresinol, *m*/*z* 357.1323) and five diterpene not identified in the specie (Figure 3).

### 2.2. Antioxidant Activity Determined by Thin-Layer Chromatography (TLC)

**Figure 4:**
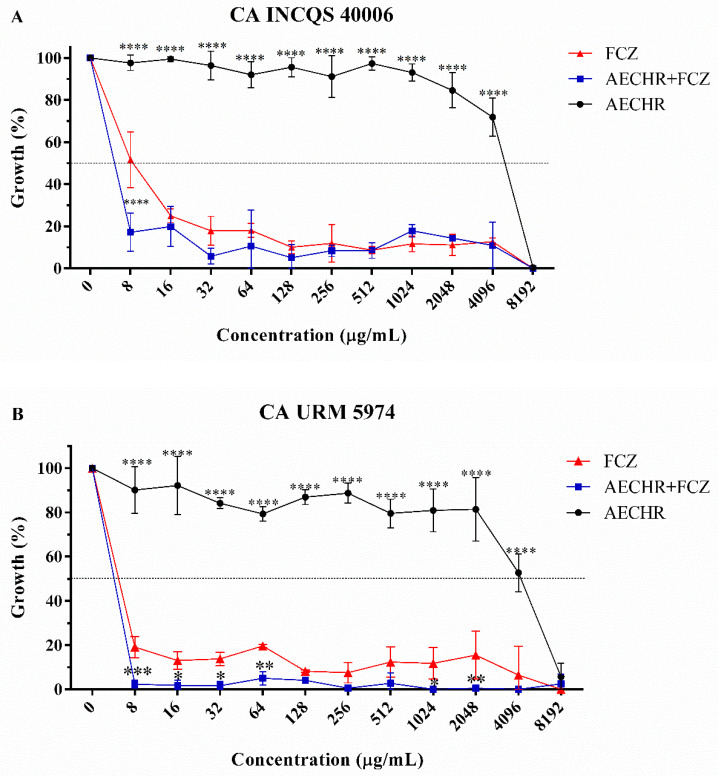
Cell viability curve of *Candida albicans* strains ((**A**), CA INCQS 40006; (**B**), CA URM 5974) treated with the aqueous extract of *C. heliotropiifolius* roots (AECHR) or fluconazole (FCZ). (**A**)—**** = *p* < 0.0001; (**B**)—* = *p* < 0.05; ** = *p* < 0.01; *** = *p* < 0.001.

Thin-layer chromatography demonstrated the presence of phenolic substances with varied polarities, the most polar being located at the bottom of the plate (Figure 4A), consistent with the dark blue colors resulting from the reaction with FeCl3, as well as the positive controls quercetin (Spot 1 in Figure 3A) and gallic acid (Spot 2 in Figure 3A). The yellow-colored bands in Figure 3B indicates DPPH radical scavenging sites, as do the positive controls quercetin (Spot 1 in Figure 3B) and gallic acid (Spot 2 in Figure 3B).

According to Formagio et al. [34] and Hidalgo, Nunomura, and Nunomura [35], plant extracts that have blue-colored substances revealed by FeCl_3_ and yellow colors revealed by DPPH in chromatoplates are phenolic substances with antioxidant action. Thus, the results obtained in the present study indicate that the analyzed extract presented polar and non-polar phenolic substances with antioxidant action.

It is also worth noting that the use of gypsum/starch (1:1) as a fixed phase in glass chromatoplates was an alternative to the use of silica gel, widely used in CCD, as well as prefabricated plates with aluminum sheets coated with silica gel, which are significantly costly [36].

### 2.3. Antifungal Effects of C. heliotropiifolius against C. albicans

Table 3 shows the IC_50_ values of the extract and fluconazole when tested either in combination or separately against *Candida albicans* strains. Note that the extract presented IC_50_ values higher than that of the reference antifungal against both *C. albicans* strains. However, the combination of the extract with fluconazole resulted in a synergistic effect against the INCQS 40006 strain, thus reducing the IC_50_ of the standard antifungal drug.

As observed in the cell viability curve (Figure 4), the extract did not show significant antifungal activity, inhibiting the growth of the strains only at the highest concentration tested. On the other hand, the curve of fluconazole indicated growth inhibition at all tested concentrations, which was potentiated by the combination with the extract ata concentration of 8 μg/mL, especially against the CA INCQS 40006 strain, indicating a possible modulating effect on antifungal resistance. Additionally, similar effect was observedat several points of the CA URM 5974 growth curve.

### 2.4. Minimum Fungicidal Concentration (MFC)

The CFM was defined as the lowest concentration at which no colony growth was observed after 24 h. In assays using the CA INCQS 40,006 strain, groups treated with the extract, fluconazole, and the combination of both showed a CFM ≥ 16,384 μg/mL (not shown). On the other hand, in assays using the CA URM 5974 strain, only fluconazole presented a CFM lower than 16,384 μg/mL (8192 μg/mL), indicating the absence of antifungal activity potentiation under these experimental conditions.

### 2.5. Effects of Croton heliotropiifolius Extract on Fungal Morphology

Morphological analyses were carried out to investigate the effect of the extract on the dimorphism of *Candida albicans* strains. Therefore, the extract was tested at the following concentrations based on the matrix concentration (MC) value: 8192 μg/mL (MC/2), 4096 μg/mL (MC/4), and 1024 μg/mL (MC/16).

**Figure 5:**
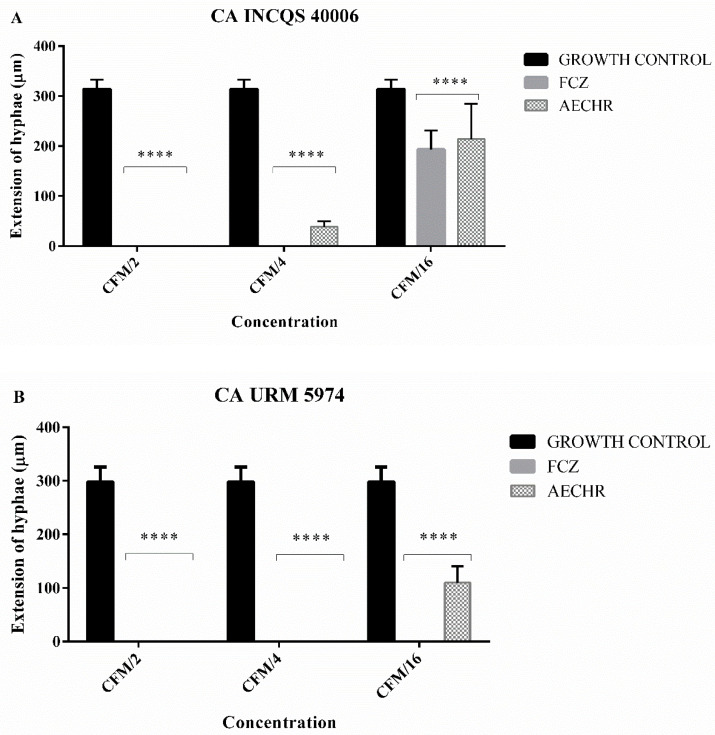
Effects of *Croton heliotropiifolius* extract on the morphological transition *of C. albicans*. The absence of bars occurs due to complete inhibition of filamentous structures. The extract and fluconazole inhibited the morphological transition at some concentrations. **A**—**** = *p* < 0.0001; **B**— **** = *p* < 0.0001.

Fluconazole was tested at the same concentrations, except against the CA URM 5974 strain, due to differences in matrix concentration: 4096 μg/mL (MC/2), 1024 μg/mL (MC/4), and 512 μg/mL (MC/16). It was demonstrated that the extract, at the highest tested concentration, inhibited the dimorphism of the CA INCQS 40006 strain, for which no hyphae emission was observed (Figure 5A). At other concentrations, the extract did not inhibit dimorphism but decreased the length of these virulence structures compared with the control group. On the other hand, fluconazole did not prevent hyphae emission except at the lowest tested concentration.

Regarding the CA URM 5974 strain (Figure 5B), more promising results were observed with fluconazole and the extract. The extract completely inhibited dimorphism at the concentrations of 8192 μg/mL (CM/2) and 1024 μg/mL (CM/4) and decreased the length of these structures at the concentration of 512 μg/mL (CM/16) compared with the control group.

### 2.6. Antiparasitic and Cytotoxic Effects of C. heliotropiifolius

Regarding the antiparasitic activity of the *C. heliotropiifolius* extract, it is possible to notice that the extract had low antiparasitic activity against *T. cruzi* epimastigotes, in addition no significant effects against *L. braziliensis* and *L. infantum*. Accordingly, the extract presented low cytotoxicity at higher concentrations against mouse fibroblasts (Table 4). The IC_50_ value against parasites and fibroblasts was 1000 μg/mL.

## 3. Discussion

*Croton* species are used in traditional Brazilian medicine to treat infections, inflammation, and gastrointestinal disorders such as diarrhea and dysentery [37], which can be caused by *Candida* sp infections. Accordingly, some studies have already reported the antifungal activity of some species of this genus against *Candida* strains [38,39,40,41,42,43,44]. Furthermore, previous studies have demonstrated the antifungal activity of both the ethanolic extract and isolated compounds of *Croton heliotropiifolius* against *Candida albicans* strains [45], obtaining IC_50_ values even lower than those observed in the present study.

When investigating the chemical composition of the ethanol extract from the leaves of *C. heliotropiifolius*, Alencar et al. [46] also observed the presence of flavonoids, including quercetin. In addition, three of the compounds identified in the present study (quinic acid, hydroxy-benzoic acid derivative and pinoresinol) were also registered in the work by Kumar et al. [28]. Other compounds identified in this research were also elucidated in studies using liquid chromatography in the analysis of species of the same genus, including citric acid [30], Kaempferol-*O*-glucoside [32], malic acid, quinic acid, and quercetin-O-glycoside [29]; the latter was also identified by Nascimento et al. [31].

The antioxidant activity of plants belonging to the genus *Croton* has been widely investigated [47]. In this context, the antioxidant properties of the ethanolic extract of *C. heliotropiifolius* leaves were recently demonstrated by Rodrigues et al. [48] and Aquino et al. [49], who attributed such activity to the presence of phenolic compounds, including some compounds identified in the present study. Such compounds were also identified in the ethanolic extract of the stem bark [50], as well as in a methanolic extract of the leaves [51] of *C. heliotropiifolius*, also correlating with the antioxidant properties of this species. In addition to the antioxidant activity, lignans such as pinoresinol found in this study have antibacterial activity and may also reduce the risk of breast cancer. When included in the diet, their consumption was associated with a low incidence of atherosclerosis, cardiovascular disease, and some cancers, and also demonstrated activity against some viruses [52,53].

Carboxylic acids have been shown to have antimicrobial properties [54], with significant antifungal activity against *C. albicans* [55]. However, Queiroz et al. [45] demonstrated, in a test with a carboxylic acid isolated from *C. heliotropiifolius*, failed to show activity against *C. albicans*. Thus, it is hypothesized that the antifungal effects demonstrated in this study are due to the presence of flavonoids, a wide range of metabolites with well-established antimicrobial activity. Serpa et al. [56] observed synergistic activity between flavonoids and fluconazole against *Candida* infections. For the flavonoids quercetin and catechin, several activities have been verified, such as antioxidant, anti-inflammatory, anticancer, antiparasitic, antiviral, biofilm reduction, and protection of the cardiovascular, renal, and hepatic systems [57]. Additionally, Araújo et al. [58] found terpenes to be the main compounds of the essential oil of *C. heliotropiifolius*, suggesting that these molecules may be significantly responsible for the antimicrobial activity of this species. Terpenoids may have a role in the chemoprevention of colon cancer [59].

*Candida albicans* is an opportunistic fungus that can cause invasive infections in immunocompromised individuals [11]. Worryingly, increased resistance to antifungal drugs threatens the effectiveness of treatments used to combat infections caused by this microorganism [60]. In this context, a study analyzing antifungal resistance in infections by *Candida* species, including *C. albicans*, in Brazilian hospitals recorded high rates of resistance to conventional antifungal agents such as fluconazole (88.89%), miconazole (66.67%), nystatin (55.56%), and amphotericin B (50.00%) [61]. Similar studies found resistance rates to fluconazole in *C. albicans*, ranging between 61% and 100% [62,63]. Fluconazole belongs to the class of azoles, whose mechanism of action involves the inhibition of lanosterol 14-α-demethylase, which catalyzes the synthesis of ergosterol (the main sterol in the fungal cell membrane), resulting in altered membrane permeability and inhibition of replication and hyphae emission, leading to the accumulation of 14-α-methyl-3,6-diol, a toxic sterol [31,64].

Resistance to this class of antifungal agents mainly involves the following mechanisms: activation of efflux pumps, decreased sensitivity preventing the binding of azoles, and ERG3 gene mutation reducing the accumulation of 14-α-methyl-3,6-diol [65,66].

Given the high rates of antifungal resistance, research has been conducted to identify natural products that are capable of increasing the effectiveness of conventional antifungal agents against *Candida* infections [67,68]. Accordingly, different classes of natural products have been shown to be able to modulate resistance to azoles [67]. The present study demonstrated the modulating effects of the ethanolic extract of *C. heliotropiifolius* roots, corroborating previous work that found similar effects for plants of the same genus against *Candida* strains [69,70].

The morphological transition in *C. albicans* is a virulence factor crucially involved in the invasiveness of this microorganism [71]. It is important to emphasize that previous research has identified natural products that are capable of inhibiting such phenomena [72]. In this study, *C. heliotropiifolius* extract significantly inhibited hyphae emission in both the standard strain (CA INCQS 40006) and the clinical isolate (CA URM 5974) of *Candida albicans*, indicating the inhibition of fungal dimorphism, with probable effects on the virulence of these strains, which should be further investigated using infection models [68,72,73,74]. 

Based on previous studies, it is reasonable to assume that this effect may be due to the presence of components such as carboxylic acids, terpenes [55,58], and phenolic compounds [69]. However, further research is required to investigate the action of isolated compounds.

With regard to the evaluation of anti-kinetoplastidae activity, the results of the present study differ from those found by Alencar et al. [46], who used an ethanolic extract obtained from the leaves of *C. heliotropiifolius*. These authors observed both leishmanicidal and trypanocidal effects, but with high cytotoxicity against fibroblasts. On the other hand, Ramos et al. [75] demonstrated that hexane extracts obtained from both the leaves and stem of this plant showed moderate cytotoxicity. In this study, we demonstrated that the root extract does not have significant antiparasitic effects. On the other hand, it has the advantage of presenting low toxicity. Thus, the potential of *C. heliotropiifolius* in the treatment of fungal skin infections is highlighted, both for its anti-*Candida* effects and its low toxicity against fibroblasts, cells with important roles in immunity and tissue regeneration [46,76].

## 4. Materials and Methods

### 4.1. Botanical Material

Healthy roots of *Croton heliotropiifolius* Kunth were collected in an Environmental Protection Area belonging to the “Chapada do Araripe” Crato, southern Ceará, Brazil (07°12′51.169″ S; 39°31′30.75″ W; 922 m altitude). The species collection was authorized by the Biodiversity Authorization and Information SystemSisBio (registry number 64011-1), and its use in the present study was registered in the National System for Management of Genetic Heritage and Associated Traditional Knowledge (registry number A31E860). The botanical material was identified by Ana Cleide Morais Mendonça de Alcantara, and a voucher specimen was registered at the Herbarium Caririense Dárdano de Andrade Lima of the Regional University of Cariri – URCA (registry number 13.554).

### 4.2. Extract Preparation

The aqueous extract was prepared using 500 g of *C. heliotropiifolius* roots. After collection, the plant material was washed in running water and cut into small pieces of approximately 1 cm. The decoction was then prepared using 266.7g of roots to 4 L of water, according to the methodology proposed by Matos [77]. The extract was dehydrated by spray-drying using a Mini-spray dryer (model MSDi 1.0, Labmaq do Brasil, Ribeirão Preto, Brazil) with a 1.2 mm sprinkler nozzle, under the following operating conditions: (a) flow control: 500 mL/H; (b) inlet temperature: 130 ± 2 °C; (c) outlet temperature: 76 ± 2 °C; (d) atomizing airflow: 45 L/min; (e) blower flow: 1.4 m^3^/min [78]. At the end of dehydration, 1.109 g of powdered extract was obtained and stored under refrigeration at 10 °C.

### 4.3. Phytochemical Analysis

#### 4.3.1. Qualitative Analysis

A preliminary qualitative analysis of the extract aiming to identify the main classes of secondary metabolites was carried out [79]. Briefly, after the addition of specific reagents, the presence of different classes of metabolites wasdemonstratedby the change in color or formation of a precipitate, allowing qualitative identification of the presence of the following compounds: alkaloids, anthocyanins, anthocyanidins, catechins, coumarins, steroids, phenols, flavones, flavanones, flavonols, flavanonols, leucoanthocyanidins, tannins, triterpenoids, and xanthones. Initially, 1000 mg of the crude extract was dissolved in 100 mL of distilled water. Subsequently, 3 mL aliquots of these solutions were added to test tubes, to which specific reagents were added. All experiments were performed in triplicate.

#### 4.3.2. Ultra-Efficiency Liquid Chromatography Coupled with a Quadrupole/Time-of-Flight System (UPLC-QTOF)

The chemical composition of the extract was determined using the Acquity UPLC system (Waters Corporation, Milford, MA, USA) coupled with a Quadrupole/Time of Flight system (QTOF). Chromatographic runs were performed on a Waters Acquity UPLC BEH column (150 × 2.1 mm, 1.7 µm) injected with 5 µL of the extract solution, with the temperature adjusted to 40 °C. The binary gradient elution system consisted of 0.1% formic acid in water (A) and 0.1% formic acid in acetonitrile (B), with a linear gradient from 2% to 95% of B (0–15) min) and a flow rate of 0.3 mL/min [80].

The ESImode was acquired in the range of 110–1180 Da, with the source temperature fixed at 120 °C, a desolvation temperature of 350 °C, a desolvation gas flow of 500 L/h, an extraction cone of 0.5 V, and a capillary voltage of 2.6 kV; the acquisition mode was MSE, with the instrument controlled by the Masslynx 4.1 software (Waters Corporation, Milford, MA, USA). The precise molecular formula and mass assignments were obtained with MassLynx 4.1 software (Waters Corporation, Milford, MA, USA). The data were compared with those described in the literature at the level of the botanical family of the species. Peak identification was determined by m/z values.

### 4.4. Analysis of Antioxidant Activity by Thin-Layer Chromatography (TLC)

This analysis was carried out based on the methodology proposed by Soler-Rivas et al. [81], with some adaptations. The plant extract was analyzed in triplicate by thin-layer chromatography (TLC) using quercetin and gallic acid as positive standards for comparison (1 mg/mL in methanol). As an adaptation of the method, chromatographic plates were prepared using glass plates (10× 5× 0.3 cm) as a support, and a mixture of gypsum and corn starch (1:1) was used as a stationary phase [82,83]. Aliquots of 20 μL of each sample were added to the plates along with the positive controls and the elution system: chloroform/ethanol (9:1). After drying at room temperature (30 ± 2 °C), the plates were sprayed separately with specific developers, as follows: (a) a phenolic compound developer: diluted ferric chloride solution (FeCl_3_, 2%), resulting in a dark blue color; (b) an antioxidant compound developer: a 0.5% solution of the 2,2-diphenyl-1-picrylhydrazyl (DPPH) radical in methanol, resulting in the appearance of yellowish spots on a purple background; (c) an antioxidant compound developer with reducing potential: a mixture (1:1) of aqueous solutions of K_3_[Fe(CN)_6_](1%) and FeCl_3_ (0.1%), resulting in dark blue spots (Prussian blue).

### 4.5. Antifungal Potential Analysis

#### 4.5.1. Cell Cultures

The antifungal activity of the extract was evaluated against a standard strain (CA INCQS 40006) and a clinical isolate (CA URM 5974) of *Candida albicans*. These strains were inoculated in Sabouraud Dextrose Agar (SDA, KASVI, Laboratorios Conda S.A., Spain) and incubated at 37 °C for 24 h. After this period, culture aliquots were transferred to test tubes containing 3 mL of saline solution (0.9% sodium chloride), and the concentrations were adjusted by comparing the inoculum turbidity with 0.5 on the McFarland scale [84] Microdilutions were performed using doubly concentrated Sabouraud dextrose broth (HiMedia, Mumbai, India). Depleted potato dextrose agar (PDA) (Becton Dickinson Rowa France, Le Pont de Claix, France) with added bacteriological agar was used for micromorphological analysis. The CA INCQS 40006 strain was obtained from the Reference Collection of Microorganisms in Sanitary Surveillance (CMRVS) of the National Institute of Health Quality Control (INCQS, FIOCRUZ), while the CA URM 5974 strain was obtained from the University Recife Mycology (URM) library of the Federal University of Pernambuco.

#### 4.5.2. Drugs and Reagents

The extract was dissolved in dimethylsulfoxide (DMSO, Merck, Darmstadt, Germany) and diluted in sterile water at a test concentration of 16,384 μg/mL [85]. The antifungal drug fluconazole (Capsule – Prati, Donaduzzi and CIA LTDA, Toledo, Brazil) was diluted in water to the test concentration (16,384 μg/mL) and used as a reference control drug.

#### 4.5.3. Determination of Intrinsic Antifungal Effect

The intrinsic antifungal effect was determined by the microdilution method in 96-well plates. The aqueous extract and fluconazole (Capsule – Prati, Donaduzzi and CIA LTDA, Toledo, Brazil) was serially diluted at concentrations ranging from 8192 to 8 μg/mL in a 96-well plate containing the inoculum and SDB medium [86]. The plates were incubated at 37 °C for24 h, then the readings were performed at a wavelength of 630 nm in a spectrophotometer (Thermoplate). The values obtained from these readings were used to elaborate the cell viability curve from which the IC_50_ values were determined [87]. Wells containing the vehicle (0.9% saline solution) or the medium were used as sterilecontrols. These experiments were performed in triplicate.

#### 4.5.4. Minimum Fungicidal Concentration (MFC) Determination

The tip of a sterile swab was inserted into each well of the plate used in the microdilution test and used to generate a subculture in a Petri dish, at the bottom of which, a guide plate was fixed. After 24 h of incubation, the plates were inspected for the formation of *Candida albicans* colonies [88]. The MFC was defined as the lowest concentration at which no growth of fungal colonies was observed.

#### 4.5.5. Analysis of Antifungal Resistance Modulation

To assess the ability of the extract to modulate antifungal resistance, we investigated whether its association with the standard drug fluconazole would result in enhanced antifungal activity. Therefore, the extract was used in a subinhibitory concentration in relation to the matrix concentration (CM/16), where CM is equivalent to 16,384 μg/mL [89]. Plates containing the medium, inoculum, and the extract were added to fluconazole at concentrations ranging from 8 to 8192 µg/mL. The plates were incubated at 37 °C for 24 h in an oven, and then readings were performed as previously described.

#### 4.5.6. Effects of *C. heliotropiifolius* Extract on Fungal Morphology

Chambers containing sterile slides and dilution-depleted PDA medium were added with different extract concentrations (CM/2, CM/4, and CM/16). On the solid medium, two parallel streaks were drawn and covered with a sterile coverslip. The chambers were incubated at 37 °C;24 h later, they were analyzed by light microscopy (AXIO IMAGER M2-3525001980, ZEISS, Germany) at 200×magnification [90,91]. The size of the hyphae was determined using Zen 2.0 software. For this purpose, five photos of each slide were taken in random fields. The results were expressed as the mean size of 25 hyphae analyzed on each slide [92]. Untreated and fluconazole-treated groups were used as experimental controls.

### 4.6. Antiparasitic Activity Analysis

#### 4.6.1. Anti-*Leishmania* Activity Determination

The analysis of anti-*Leishmania* activity was evaluated against *Leishmania braziliensis* (MHOM/BR/75/M2903) and *Leishmania infantum* (MCAN/ES/92/BCN 83) promastigotes cultured at 22 °C in Schneider’s Drosophila medium supplemented with 20% FBS, as described by Mikus and Steverding [93]. Briefly, promastigotes were cultivated in microdilution plates at a concentration of 2.5 × 10^5^ parasites/well, treated with the extract (250 to 1000 µg/mL) in a final volume of 200 µL, and incubated at 26 °C for 48 h. After incubation, 20 μL of resazurin solution was added to each well. After reaction, readings were taken at 570 and 595 nm in a spectrophotometer. The median lethal concentration (LC_50_) was determined and the antipromastigote percentage (%AP) was calculated using the following equation:

$$\%\mathrm{AP}=\frac{\mathrm{AE}-\mathrm{AEB}}{\mathrm{AC}-\mathrm{ACB}}\times 100$$

where AE is the absorbance of the experimental group, AEB is the compound blank, AC is the absorbance of the control group, and ACB is theculture medium blank.

#### 4.6.2. *In Vitro* Susceptibility of *Trypanosoma cruzi* Epimastigotes

The trypanocidal effects of the extract were evaluated using *Trypanosoma cruzi* (Clone CL-B5) epimastigotes [94] stably transfected with the lacZ gene of *Escherichia coli* β-galactosidase, provided by Dr. F. Buckner through the Gorgas Memorial Institute, Panama. Parasites were cultured at 28 °C in LITB (Difco, Detroit, MI, USA), supplemented with 10% FCS (Gibco, Carlsbad, CA, USA), penicillin (Ern, SA, Barcelona, Spain), and streptomycin (Reig JofreSA, Barcelona, Spain) [95].

Epimastigotes that did not reach the stationary phase were seeded at a concentration of 1 × 10^5^ cells/mL in microdilution plates, followed by the addition of the extract (250–1000 μg/mL) ata final volume of 200 μL per well. The plates were incubated at 28 °C and, after 72 h, 50 μL of CPRG solution was added at a final concentration of 200 µM. Following this step, the plates were incubated at 37 °C for 6 h, and then readings were taken at 595 nm. The LC_50_ and the percentage of antiepimastigoteactivity (%AE) were calculated as previously described.

#### 4.6.3. *In Vitro* Cytotoxicity Study

To assess the cytotoxicity of the extract on mouse fibroblast cells, the fibroblast cell line NCTC 929 (ATCC, Washington, District of Columbio, USA) was used, following the colorimetric method described by Rolón et al. [96]. Cells were cultured in RPMI medium (Roswell Park Memorial Institute, Sigma-Aldrich, St. Louis, MO, USA), supplemented with 10% fetal bovine serum (inactivated by heat at 56 °C for 30 min), penicillin G (100 U/mL, Sigma-Aldrich, St. Louis, MO, USA), and streptomycin (100 mg/mL; Sigma-Aldrich, St. Louis, MO, USA) and maintained at 37 °C in a 5% CO_2_ atmosphere.

NCTC 929 cells were seeded (3 × 10^4^ cells/well) in flat-bottomed microdilution plates (96 wells) containing 100 µL of RPMI in each well and cultured at 37 °C in a 5% CO_2_ atmosphere overnight. The medium was then replaced by 200 µL of a new medium, where the extracts were diluted extracts (250–1000 µg/mL). After an incubation period of 24 h, 20 µL of a resazurin solution (2 mM) was added to each well; 3 h later, readings were performed at 490 and 595 nm. Tests were performed in triplicate, using the experimental controls as described above. The percentage of cytotoxicity was determined using the following equation:

$$\%C=\frac{A_{570}\times 117,216-A_{595}\times 80,586\;\left(\mathrm{test}\;\mathrm{sample}\right)}{A_{570}\times 117,216-A_{595}\times 80,586\left(\mathrm{control}\right)}\times 100$$

where %C corresponds to the percent toxicity of the extract; A_570_ and A_595_ represent the optical density values at 570 and 595 nm, respectively;and the values 80.586 and 117.216 are the molar extinction coefficients for resazurin corresponding to the respective absorbances.

### 4.7. Statistical Analysis

Data were checked for normal distribution and then analyzed by one-way ANOVA comparing the values of each extract concentration, point by point, viaBonferroni’s post hoc test. The IC_50_ values were obtained by non-linear regression from the interpolation of unknowns from standard growth curves as a function of extract concentration, expressed in μg/mL. These analyzes were performed using Graphpad Prism software, version 6.0 (Graphpad Software, Inc, San Diego, CA, USA).

## 5. Conclusions

The data obtained in the present study revealed that the aqueous extract of *C. heliotropiifolius* roots is composed by phenolic compounds with antioxidant activity, besides presenting a considerable variety of secondary metabolites, such as alkaloids, phenols, flavones, flavonols, flavanones, and xanthones. 

Although intrinsic fungicidal activity was not demonstrated, the extract was able to potentiate the activity of fluconazole, indicating a possible modulation of antifungal resistance. The extract inhibited fungal dimorphism of both the standard strain and the clinical isolate of *C. albicans*, which might impact both the virulence and pathogenicity shown by these microorganisms.

Despite the low cytotoxicity against fibroblasts, the effects demonstrated against *T. cruzi*, *L. braziliensis*, and *L. infantum* do not encourage the use of the extract as an antiparasitic, although tests with isolated components are essential to better characterize the pharmacological activities demonstrated in the present study. In conclusion, *C. heliotropiifolius* has antioxidant and antifungal properties associated with low cytotoxicity, encouraging further comprehensive studies to evaluate its potential for the treatment of fungal skin infections.

## Figures and Tables

**Table 1:** Special metabolite classes identified in the aqueous extract of *C. heliotropiifolius* roots.

SAMPLE	Special Metabolite Classes (SMC)									
	SMC 1	SMC 2	SMC 3	SMC 4	SMC 5	SMC 6	SMC 7	SMC 8	SMC 9	SMC 10
PRESENCE	+	-	+	-	-	+	-	-	-	+

AECHR - aqueous extract of *C. heliotropiifolius* roots; SMC1—phenols; SMC2—tannins; SMC3—flavones, flavonols, and xanthones; SMC 4—anthocyanins and anthocyanidins; SMC5—leukoanthocyanidins and catechins; SMC6—flavanones; SMC7—flavanonols; SMC8—steroids; SMC9—triterpenoids; SMC10—alkaloids; (+)—present; (-)—absent_._

**Table 2:** Compounds identified in the aqueous extract of *Croton heliotropiifolius* roots in the negative mode.

Peak	RT min^a^	[M–H]^−^ Obs.^b^	[M–H]^−^ Calc.^b^	Ions (MS/MS)	Emp. Formula	Ppm (error) ^c^	Putative Name	Ref.
1	2.78	272.9554	272.9551	-	C_8_H_2_N_2_O_7_Cl	1.1	Unknown	-
2	3.15	191.0549	191.0556	-	C_7_H_11_O_6_	−3.7	Quinic acid	[28,29]
3	3.45	133.0141	133.0137	115	C_4_H_5_O_5_	2.3	Malic acid	[29]
4	3.78	191.0185	191.0192	111	C_6_H_7_O_7_	−3.7	Citric acid	[30]
5	4.50	463.0894	463.0877	301	C_21_H_19_O_12_	3.7	Quercetin -*O*-glucoside	[29,31]
6	4.89	457.1339	457.1346	171	C_20_H_25_O_12_	0.3	Unknown	-
7	5.07	447.0914	447.0927	285	C_21_H_19_O_11_		Kaempferol-*O*-glucoside	[32]
8	5.20	253.0347	253.0348	137	C_11_H_9_O_7_	−0.4	Hydroxy- benzoic acid derivative	[28]
9	5.56	335.2234	335.2222	-	C_20_H_11_O_4_	3.6	Diterpene	-
10	5.75	357.1323	357.1338	327, 313	C_20_H_21_O_6_	−4.2	Pinoresinol isomer	[28]
11	6.00	335.2220	335.2222	-	C_20_H_31_O_4_	−0.6	Diterpene	-
12	6.35	301.0359	301.0348	-	C_15_H_9_O_7_	3.7	Quercetin	[29,31]
13	6.45	269.0450	269.0450	-	C_15_H_9_O_5_	0.0	Apigenin	[33]
14	6.71	303.1958	303.1960	-	C_19_H_27_O_3_	-0.7	Diterpene	-
15	6.96	303.1950	303.1960	-	C_19_H_27_O_3_	−3.3	Diterpene	-
16	7.86	317.2115	317.2117	-	C_20_H_29_O_3_	−0.6	Diterpene	-

^a^ RT, retention time; ^b^ molecular ion observed and calculated; ^c^ ppm, parts per million.

**Table 3:** Half-maximal inhibitory concentration (IC_50_) of *C. heliotropiifolius* extract and fluconazole against *C. albicans* strains.AECHR—aqueous extract of *C. heliotropiifolius* roots; AECHR*—subinhibitory concentration of the extract (1024 μg/mL); FCZ—fluconazole; INCQS—National Institute of Quality Control in Health; URM—University Recife Mycology. (*p* < 0.0001).

STRAIN	AECHR IC_50_ (μg/mL)	FCZ IC_50_ (μg/mL)	AECHR* + FCZ IC_50_ (μg/mL)
INCQS 40006	5459.3	8	1.7
URM 5974	4385.3	1.7	1.6

**Table 4:** Antiparasitic and cytotoxic effects of the *Croton heliotropiifolius* extract.

AECHR (μg/mL)	%AE *T. cruzi*	%AP *L. braziliensis*	%AP *L. infantum*	%Cit. Fibroblasts
1000	33.67 ± 0.49a	0.00 ± 1.06a	0.00 ± 0.29a	14.66 ± 7.35
500	13.12 ± 0.70b	0.00 ± 0.02a	0.00 ± 0.31a	-
250	8.66 ± 0.95c	0.00 ± 7.99a	0.00 ± 1.15a	-

Legend: %AE: percentage of antiepimastigote activity; %Cit: percentage of cytotoxicity. Different letters in the same column indicate statistical significance by Tukey’s test with reliability at a 95% confidence level.

## Data Availability

The data present in this study are available on request from the corresponding autor.
